# Innovative Technologies: Reverse Osmosis Moves Forward

**DOI:** 10.1289/ehp.113-a25a

**Published:** 2005-01

**Authors:** Carol Potera

As drought and growing populations cause water supplies to dwindle in areas around the world, reclaimed wastewater offers a possible solution. Indeed, some communities in California already use reclaimed wastewater to irrigate crops, water golf courses, and augment freshwater aquifers to block saltwater intrusion. Critics are concerned about the potential health hazards of the pharmaceuticals, hormones, and other contaminants that even treated wastewater has been shown to contain. But recent research reveals that the process of reverse osmosis may remove some of these contaminants.

As described in the 12 March 2004 issue of the *Journal of Chromatography A*, Joel Pedersen, an environmental chemist at the University of Wisconsin–Madison, and his colleagues used gas chromatography–mass spectrometry to look for 19 compounds in effluent samples collected from reclaimed wastewater plants in California. They found detectable concentrations for 13, including food preservatives, painkillers, oral contraceptive hormones, and prescription medications. However, at the 228th American Chemical Society meeting held in Philadelphia in August 2004, Pedersen further reported that gas chromatography confirmed all 13 compounds to have been eliminated at two pilot plants testing reverse osmosis for contaminant removal.

Nonetheless, Pedersen cautions that it’s too early to recommend that all reclaimed wastewater facilities employ reverse osmosis. “This is a case where the analytical chemistry is ahead of the toxicology,” he says.

“Little is known about the toxicity of trace concentrations of these compounds,” agrees Shane Snyder, project manager of research and development at the Southern Nevada Water Authority (SNWA) in Las Vegas. Snyder has monitored the flow of treated wastewater effluent into nearby Lake Mead since 1997. He says fish in Las Vegas Bay are the healthiest in all of Lake Mead because they thrive on nutrients in the effluent. Snyder and colleagues at the U.S. Fish and Wildlife Service are writing a paper on this topic.

Often used to remove salts, reverse osmosis requires electricity to pump water through semipermeable membranes. “A lot of work is involved to perform reverse osmosis correctly,” says Pedersen. “Large-scale reverse osmosis may not be economically feasible in some areas.” Salts, contaminants, and biofilms can clog the pores of membranes, raising maintenance costs.

Still other costs can make the process prohibitively expensive for inland cities in particular. Reverse osmosis generates brine. While coastal California wastewater facilities dump brine into the ocean, inland facilities must heat the brine to evaporate the water, then dispose of the dry salt in a landfill. “The cost of brine disposal is often more expensive than the cost of reverse osmosis itself,” says Snyder. About 30% of treated water ends up as brine during reverse osmosis. That water loss “is not acceptable when you live in the desert,” Snyder says. By comparison, standard treatment results in less than 1% water loss, according Snyder.

Moreover, “reverse osmosis membranes are not infallible,” says Snyder. For instance, the carcinogen *N*-nitrosodimethylamine, a disinfectant by-product of wastewater treatment, breaches reverse osmosis membranes. However, dangerous compounds may be removed with less expensive treatments than reverse osmosis. For example, advanced oxidation methods can destroy *N*-nitrosodimethylamine.

But it’s too soon to count reverse osmosis out just yet. Newer models require less pressure to pump water through. “More efficient membranes will lower the energy costs of reverse osmosis,” Snyder predicts, “and likely make the process more cost-effective.”

## Figures and Tables

**Figure:**
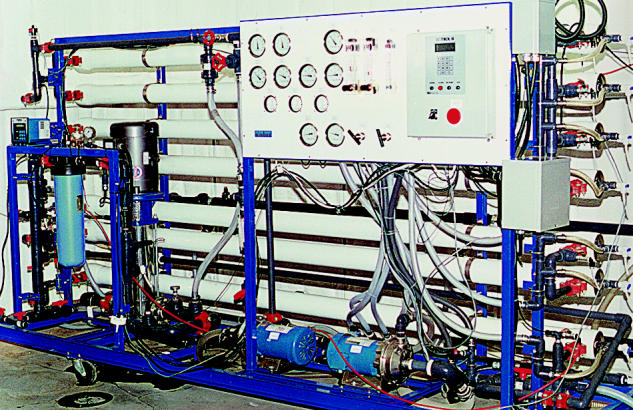
**One step back, two steps forward.** New advances in reverse osmosis may mean cleaner—and healthier—reclaimed wastewater.

